# The emerging role of fibroblast‐like synoviocytes‐mediated synovitis in osteoarthritis: An update

**DOI:** 10.1111/jcmm.15669

**Published:** 2020-07-19

**Authors:** Dafei Han, Yilong Fang, Xuewen Tan, Haifei Jiang, Xun Gong, Xinming Wang, Wenming Hong, Jiajie Tu, Wei Wei

**Affiliations:** ^1^ Institute of Clinical Pharmacology Key Laboratory of Anti‐Inflammatory and Immune Medicine Ministry of Education Anhui Collaborative Innovation Center of Anti‐Inflammatory and Immune Medicine Anhui Medical University Hefei China

**Keywords:** clinical trials, epigenetics, fibroblast‐like synoviocytes, osteoarthritis, synovitis

## Abstract

Osteoarthritis (OA), the most ubiquitous degenerative disease affecting the entire joint, is characterized by cartilage degradation and synovial inflammation. Although the pathogenesis of OA remains poorly understood, synovial inflammation is known to play an important role in OA development. However, studies on OA pathophysiology have focused more on cartilage degeneration and osteophytes, rather than on the inflamed and thickened synovium. Fibroblast‐like synoviocytes (FLS) produce a series of pro‐inflammatory regulators, such as inflammatory cytokines, nitric oxide (NO) and prostaglandin E_2_ (PGE_2_). These regulators are positively associated with the clinical symptoms of OA, such as inflammatory pain, joint swelling and disease development. A better understanding of the inflammatory immune response in OA‐FLS could provide a novel approach to comprehensive treatment strategies for OA. Here, we have summarized recently published literatures referring to epigenetic modifications, activated signalling pathways and inflammation‐associated factors that are involved in OA‐FLS‐mediated inflammation. In addition, the current related clinical trials and future perspectives were also summarized.

## 1 INTRODUCTION

Osteoarthritis (OA) is characterized by cartilage deterioration, osteophyte formation, periarticular bone sclerosis, bone resorption/formation disproportion, joint cavity alterations and synovial membrane inflammation.^1^, ^2^ OA is one of the most common joint diseases in elderly people. As there is no effective drug for OA, the current treatment is based on pain relief and symptom reduction. Appropriate exercises, weight control, physiatrics, medicine and surgery are the available methods for OA treatment, according to the guidelines of the American Academy of Orthopaedic Surgeons (AAOS) and Osteoarthritis Research Society International (OARSI).^3^, ^4^

Synovial tissue inflammation, which is known to contribute to OA development, exists in all stages of OA, even in the early stages.^5^ A lot of pro‐inflammatory cytokines, such as tumour necrosis factor (TNF), interleukin‐1 beta (IL‑1β), IL‑6, IL‑8, IL‑15, IL‑17, IL‑21, inflammatory mediators PGE_2_, NO, adipokines (visfatin, resistin) and matrix metalloproteinases (MMP‑1, MMP‑3, MMP‑9, MMP‑13) aggravate the irreversible cartilage degeneration and contribute to the progression of OA.^6^, ^7^, ^8^, ^9^ Moreover, an inflamed synovium promotes the production of adhesion molecules such as intercellular adhesion molecule‐1 (ICAM‐1), vascular cell adhesion molecule‐1 (VCAM‐1)^10^ and chemokines,^11^ which is responsible for the synovial histological changes in the OA synovium, including hypertrophy and hyperplasia accompanied by infiltrated mononuclear cells (such as monocytes and macrophages) and lymphocytes (activated T cells and B cells). Synovial inflammation also promotes synovial angiogenesis in the synovium, and this, in turn, accelerates inflammation.^12^ Furthermore, synovial inflammation facilitates the production of pro‐inflammatory and pain neurotransmitters such as nerve growth factor and bradykinin which are potential therapeutic targets for OA treatment.^13^ Inflammatory regulators and matrix degradation enzymes produced by OA synoviocytes contribute to the progression of OA.^6^, ^14^ It has been reported that synovial inflammation plays an initiator role in OA by releasing pro‐inflammatory mediators and cartilage destructive factors that induce cartilage damage, which in turn magnifies the synovial inflammation, forming a vicious cycle.^15^ Fibroblast‐like synoviocytes (FLS) constitute the predominant cellular component of the joint synovium. There are, however, two main different subsets of FLS in the synovium. The fibroblast activation protein alpha^+^ (FAPα^+^) thymus cell antigen 1^+^(THY1^+^) FLS, located in the synovial sub‐lining, selectively promotes inflammation in arthritis with little effect on the bone and cartilage destruction and the FAPα^+^ THY1^‐^ FLS, located in the synovial lining layer, which selectively promote bone and cartilage impairment with little effect on inflammation.^16^ Thus, the two non‐overlapping FLS subtypes explain the inflammatory and destructive processes that underlie OA‐FLS. Synovium‐targeted therapeutic strategies in OA may possibly prevent cartilage breakdown while alleviating other symptoms.

The concept of ‘epigenetics’ was unprecedentedly introduced in 1942 by the biologist Conrad H. Waddington, as ‘the study of heritable changes in gene expression mediated by mechanisms rather than alterations in the primary nucleotide sequence of a gene’. Nowadays, epigenetics is broadly used to refer to heritable and reversible variations in gene expression and cell functionality that are independent of the DNA sequence.^17^, ^18^ Epigenetic modifications mainly include DNA methylation, histone modifications, chromosomal remodelling and non‐coding RNAs, including microRNAs (miRNAs), and long‐non‐coding RNAs (lncRNAs). In recent years, epigenetic modifications have been shown to play an important role in the pathophysiology of OA by changing the production of pro‐inflammatory cytokines, proteins involved in apoptosis and MMPs.^19^

The nuclear factor‐kappa B (NF‐κB) family plays an important role in inflammation and/or immune responses as well as cell survival, proliferation and differentiation.^20^ In humans, the NF‐κB family comprises five members: RelA/p65, RelB, c‐Rel, NF‐κB1/p105 and NF‐κB2/p100. All NF‐κB proteins share a common structure: a conserved N‐terminal Ref‐1‐homology domain (RHD) that is vital for interacting with NF‐κB inhibitors (IκBs), forming dimers, binding to DNA and nuclear translocation.^21^, ^22^ In resting cells, the NF‐κB dimers are inactivated as they are sequestered in the cytoplasm by IκBs (IκBα, ΙκΒβ, IκBγ, ΙκΒε, Bcl‐3, p100, and p105). Once stimulated by biological or chemical signals, the IκBs become phosphorylated by IκB kinases (IKKs) leading to NF‐κB release, while the phosphorylated IκBs are degraded via the ubiquitin‐proteasome system.^21^ The uninhibited NF‐κB dimers are then permitted to translocate from the cytoplasm into the nucleus and subsequently affect the transcription of pro‐inflammatory cytokines, chemokines, adhesion molecules, growth factors and immunoregulatory genes.^23^ Activated NF‐κB regulates numerous cytokines, inflammatory regulators, transcriptions factors and MMPs.^24^

Wnt, a secreted glycoprotein of approximately 40 kDa, is highly conserved across species and plays a key role in organogenesis, morphogenesis, tumorigenesis and maintenance of stem cells.^25^ Emerging research has shown that the Wnt signalling pathway is associated with the development of OA. Activation of the β‐catenin signalling pathway in joint chondrocytes of mice leads to chondrocyte differentiation and the development of an OA‐like phenotype.^26^ This was consistent with the up‐regulated β‐catenin protein expression that was found in the knee joints of OA patients and the Frzb (the Frizzled‐related protein, also called the secreted Frizzled‐related protein 3 [sFRP‐3]) gene mutation in mice which increased their vulnerability to chemically induced OA.^27^

## 2 EPIGENETIC ALTERATIONS INVOLVED IN OA‐FLS INFLAMMATION

### 2.1 microRNAs

MicroRNAs (miRNAs) are a class of small, short, endogenous non‐coding RNAs found in eukaryotic cells, 19‐25 nucleotides in length.^28^ They regulate gene expression at the post‐transcriptional level by base‐pairing with the 3′ untranslated regions (UTRs) of their target genes, according to the extent of sequence complementarity, resulting in gene silencing by mRNA cleavage or inhibition of the mRNA translation.^29^ Emerging evidence shows that miRNAs are important epigenetic regulators that participate in the progression of OA.

**FIGURE 1:**
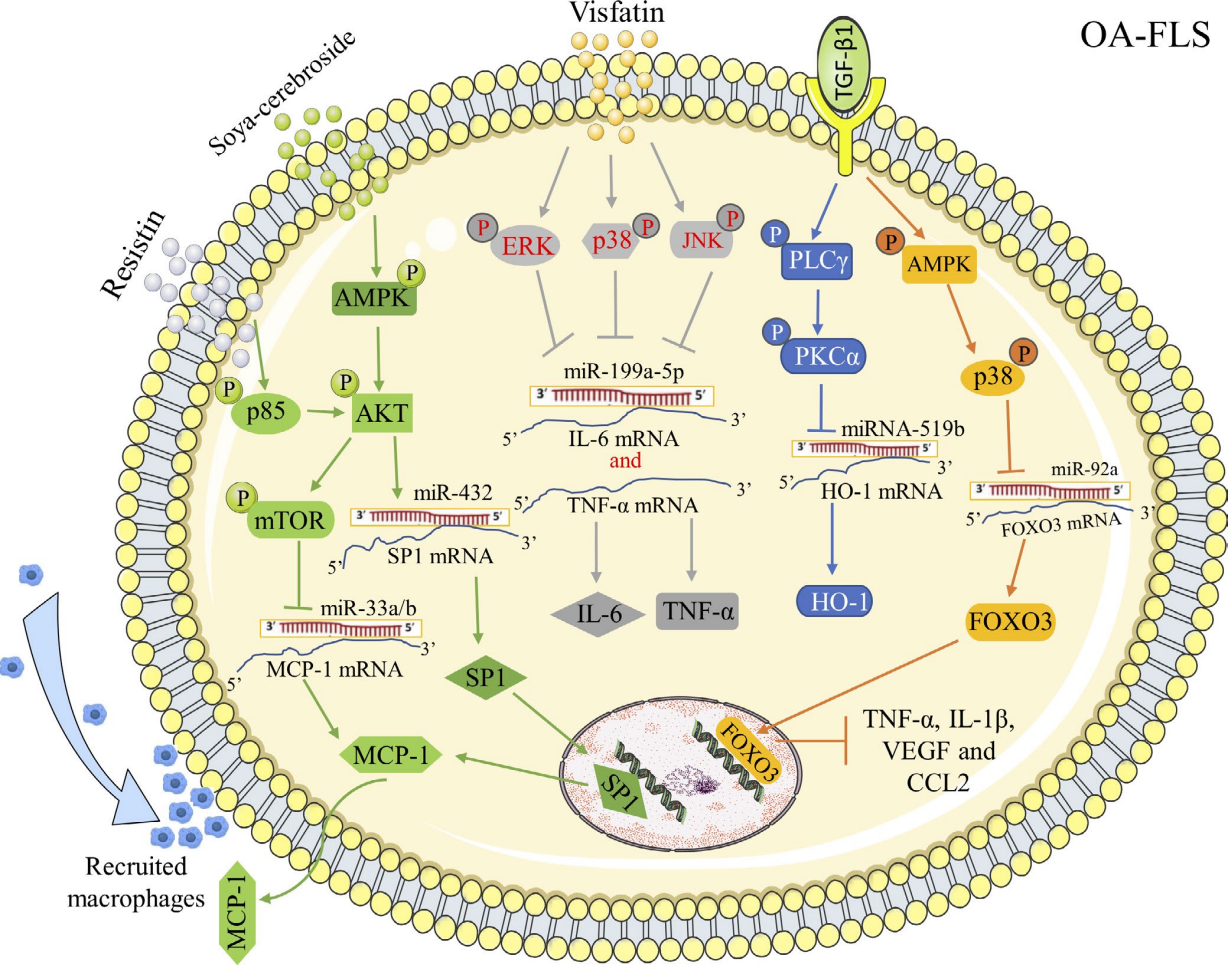
MiRNAs involved in OA‐FLS inflammation. TGF‐β1 enhanced FOXO3‐induced anti‐inflammatory effects in human OA‐FLS by suppressing miR‐92a through AMPK and p38 signalling pathway. TGF‐β1 promoted anti‐inflammatory HO‐1 generation in human OA‐FLS by blocking the synthesis of miR‐519b. Adipokine resistin enhanced the generation of MCP‐1 and promoted monocyte migration in human OA‐FLS by inhibiting miR‐33a and miR‐33b through activated PI3K/AKT/mROT signalling pathway. Adipokine visfatin enhanced IL‐6 and TNF‐α expression in human OA‐FLS by inhibiting miR‐199a‐5p via ERK, p38 and JNK signalling pathways. Soya‐cerebroside down‐regulated MCP‐1 expression in OA‐FLS by increasing miR‐432 through activated AMPK/AKT signalling pathway. PLCγ: Phospholipase Cγ; PKCα: Protein kinase C alpha; SP1: Specificity protein 1; VEGF: Vascular endothelial growth factor; MCP‐1/CCL2: Monocyte chemoattractant protein‐1/C‐C motif ligand 2

Tang *et al* focused on the role of miRNAs in the pathogenesis of OA. They observed an interaction between the transforming growth factor β1 (TGF‐β1) and miRNAs involved in OA pathophysiology. Specifically, they found that TGF‐β1 inhibited miR‐92a expression in OA‐FLS, in a concentration‐dependent manner, resulting in an increased expression of the forkhead box class O 3 (FoxO3) protein, inflammatory cytokines, such as TNF‐α, IL‐1β, vascular endothelial growth factor (VEGF), and C‐C motif ligand 2 (CCL2) through the adenosine 5'‐monophosphate (AMP)‐activated protein kinase (AMPK) and p38 signalling pathways.^12^ TGF‐β1 also increased the expression of haem oxygenase 1 (HO‐1), an inducible anti‐inflammatory enzyme,^30^ in human OA‐FLS by inhibiting miR‐519b synthesis.^31^ Moreover, adipokines, such as visfatin and resistin, were significantly up‐regulated in the serum and synovial fluid of OA‐FLS patients compared with the healthy controls.^8^, ^9^ Resistin‐stimulated monocyte chemoattractant protein‐1 (MCP‐1) expression was also up‐regulated in human OA‐FLS and contributes to monocyte migration by inhibiting miR‐33a/b via the phosphoinositide 3‐kinase (PI3K)/AKT/mammalian target of rapamycin (mTOR) pathway.^32^ Furthermore, it has been confirmed that in OA‐FLS, visfatin boosts the expression of IL‐6 and TNF‐α by inhibiting miR‐199a‐5p via the extracellular regulated protein kinases (ERK), p38, and Jun N‐terminal kinase (JNK) signalling pathways.^33^ Sara Cheleschi *et al* also confirmed significantly increased IL‐1β, IL‐6, TNF‐α, and, for the first time, IL‐17 gene expressions in cultured human OA‐FLS after stimulation with visfatin and resistin.^34^ Moreover, soya‐cerebroside, a cerebroside with anti‐inflammatory activity, isolated from *C militaris*, reduced IL‐1β‐mediated MCP‐1 production and monocyte migration by increasing miR‐432 generation through the AMPK and AKT pathways and reduced the inflammation and cartilage damage in vivo in the same manner.^35^ Stanniocalcin‐1 (STC1) was found to be the most elevated protein associated with synovium neovascularization in OA‐FLS.^36^ However, Wu et al found that STC1 expression was decreased in the OA synovial tissue and OA‐FLS cells. Furthermore, SCT1 was found to be a validated target gene of miR‐454 as SCT1 up‐regulation inhibits cell viability and IL‐6‐and IL‐8‐induced inflammatory responses but these effects could be blocked by miR‐454.^37^ The inconsistent reports regarding SCT1 expression in inflamed OA‐FLS may be due to differences in the experimental methods, such as the participating subjects, comparisons made, and functions studied. In one study, STC1 was reported to act as a promoter of neovascularization and was the most elevated gene in patient‐derived FLS from the inflamed areas compared with the FLS from normal/reactive areas. In contrast, Wu et al observed that STC1 acted as an inhibitor of inflammation and proliferation, and was found to be down‐regulated in OA‐FLS tissues and cells when compared with healthy individuals (Figure 1).

In inflamed OA‐FLS, irregularly expressed miRNAs involved in OA development bind to the 3′‐UTR of their target mRNAs, resulting in mRNA cleavage or inhibition of the mRNA translation thus negatively regulating target genes expression. Until now, miRNAs in inflamed OA‐FLS could be divided into different categories: (a) TGF‐β1‐regulated miRNAs, such as miR‐92a and miR‐519b; (b) Adipocytokines‐induced miRNAs, including miR‐33a/b and miR‐199a‐5p; (c) other abnormally expressed miRNAs, such as miR‐433 and miR‐454. In order to ease OA synovitis from the perspective of miRNAs, more favourable evidence should be provided in this field.

### 2.2 Long non‐coding RNAs

LncRNAs are a class of RNA longer than 200 nucleotides that lack protein‐coding potential. They have been confirmed to play significant roles in the progress of inflammation‐mediated diseases.^38^ Recent studies have shown that lncRNAs exert their functions via multiple mechanisms, including regulation of transcriptional patterns and protein expression, formation of endogenous small interfering RNAs (siRNAs) and natural microRNA (miRNA) sponges.

The lncRNA metastasis‐associated lung adenocarcinoma transcript 1 (MALAT1) was one of the most up‐regulated lncRNAs in OA‐FLS obtained from obese patients when compared to normal‐weight OA or non‐OA‐FLS patients. Knocking out of MALAT1 significantly decreased both the mRNA and protein production of IL‐8 in obese OA‐FLS resulting in suppressed OA‐FLS proliferation. Further pathway analysis indicated that MALAT1 participated in both the ‘inflammatory response’ and ‘inflammatory disorder’.^39^ LncRNA homeobox transcript antisense intergenic RNA (HOTAIR) was overexpressed in the FLS of a rat OA model. Silencing HOTAIR decreased inflammation (IL‐1β, IL‐6 and TNF‐α) by suppressing activation of the Wnt/β‐catenin pathway through inhibition of Wnt1 and β‐catenin production in OA‐FLS.^40^ Ectopic miR‐181c decreased osteopontin (OPN)‐induced production of IL‐6, IL‐8 and MMP‐13 by suppressing OPN mRNA production through binding to the 3’UTR of OPN in OA‐FLS. LncRNA nuclear enriched abundant transcript 1 (NEAT1), a candidate lncRNA of miR‐181c, served as a competing endogenous RNA (ceRNA) and competed with OPN for miR‐181c binding and inhibited miR‐181c expression. Silencing NEAT1 reduced inflammatory responses in OA‐FLS, while miR‐181c inhibition partially reversed the inhibitive effect of NEAT1 silencing. In addition, both OPN and NEAT1 production were increased while miR‐181c production was decreased in OA tissue compared with normal controls. Targeting NEAT1 to rescue miR‐181c and suppress OPN‐induced inflammatory responses might be a promising strategy for OA therapy.^41^

### 2.3 Other epigenetic modifications

Additional epigenetic modifications, such as DNA methylation and histone acetylation, have also been shown to play a role in OA development. DNA methylation, is one of the most well‐known epigenetic modifications, largely leading to transcription silencing, while histone acetylation is commonly associated with an activation of gene transcription.

DNA methylation occurs mainly at CpG dinucleotides involved in transforming a cytosine residue to a 5‐methylcytosine (meC) by DNA methyltransferase enzymes (DNMT1, DNMT3A, DNMT3B), resulting in transcriptional inactivation by blocking transcription factors from gene promoters.^42^, ^43^ DNA methylation participates in the development of OA by regulating the production of anti‐inflammatory or pro‐inflammatory cytokines through regulation of methylation levels in their CpG sites. Decreasing methylation of anti‐inflammatory mediators or increasing methylation of pro‐inflammatory regulators at specific CpG sites within the promoters of encoding genes by DNA methyltransferase inhibitors or DNA methyltransferase is a promising strategy in attenuating inflammation in OA. MiR‐146a was found to suppress the IL‐1 receptor‐associated kinase (IRAK1) and the TNF receptor‐associated factor 6 (TRAF6), two key molecules downstream of the TNF‐α and IL‐1β signalling pathways.^44^ MiR‐146a is deregulated in OA‐FLS due to hypermethylation of specific CpG sites in the miR‐146a regulatory regions. 5‐Aza‐2′‐Deoxycytidine (5‐AzadC, a DNA methyltransferase inhibitor) up‐regulated miR‐146a expression and decreased the binding affinity between NF‐κB transcription factors and the hypermethylated miR‐146a regulatory regions in OA‐FLS, resulting in the decreased expression of IRAK‐1, IL1β and IL‐6, which could be reversed with 5‐AzadC‐/miR‐146a inhibitor treatment.^45^ Overexpression of IL‐6 in OA‐FLS was induced by DNA hypo‐methylation in the IL‐6 promoter, increasing the DNA methylation by DNA (cytosine‐5‐)‐methyltransferase 3 alpha (Dnmt3A, a DNA methyltransferase) lead to a reduction in both the mRNA and protein levels of IL‐6.^46^

Generally, acetylated histones by acetyltransferases (HATs) refer to transcription activation by relaxing the chromatin structure, while histones deacetylated by histone deacetylases (HDACs) refer to transcription inactivation through chromatin condensation.^47^, ^48^ Deregulated HDACs and increased HATs have predominantly been identified to participate in pro‐inflammatory responses.^49^, ^50^, ^51^ In OA synovial tissue, both the total level of HDAC activity and the HDAC/HAT activity ratio are lower than the normal controls.^52^ Yang et al observed overexpressed IL‐6 in OA‐FLS and OA synovial fluid as a result of the increased H3K9/K14 and H4K12 acetylation within the IL‐6 promoter. Besides, weaker HDAC1 binding and stronger HAT1, CREB binding protein (CBP) and p300 binding were observed in the IL‐6 promoter in OA‐FLS. Moreover, anacardic acid, a histone acetyltransferase inhibitor, inhibited H3K9/K14 and H4K12 acetylation and binding of HAT1 and CBP to the IL‐6 promoter.^46^ The recruited p300 in gene promoters acetylates lysine residues at the N terminus of histones, losing the affinity between histones and DNA and making the promoter region more accessible to transcription factors and RNA polymerase II allowing for the initiation of gene transcription.^53^ Leptin concentration‐ and time dependent induced IL‐8 expression through recruiting NF‐κB/p300 to the IL‐8 promoter in OA‐FLS. The recruited p300 acetylates histone H3 and assembles RNA polymerase II to the IL‐8 promoter leading to increased IL‐8, and these effects can be antagonized by curcumin, a p300 inhibitor.^54^ Katherine et al demonstrated that 15‐deoxy‐Δ12,14‐prostaglandin J2 (15d‐PGJ2), the COX metabolite, inhibited COX‐2 expression induced by IL‐1β by suppressing H3 acetylation in the COX‐2 promoter through repressed HAT p300 recruitment without affecting HDAC in OA‐FLS.^55^ Recent research indicates that histone deacetylases (HDACs) participate in regulating miRNA expression.

**FIGURE 2:**
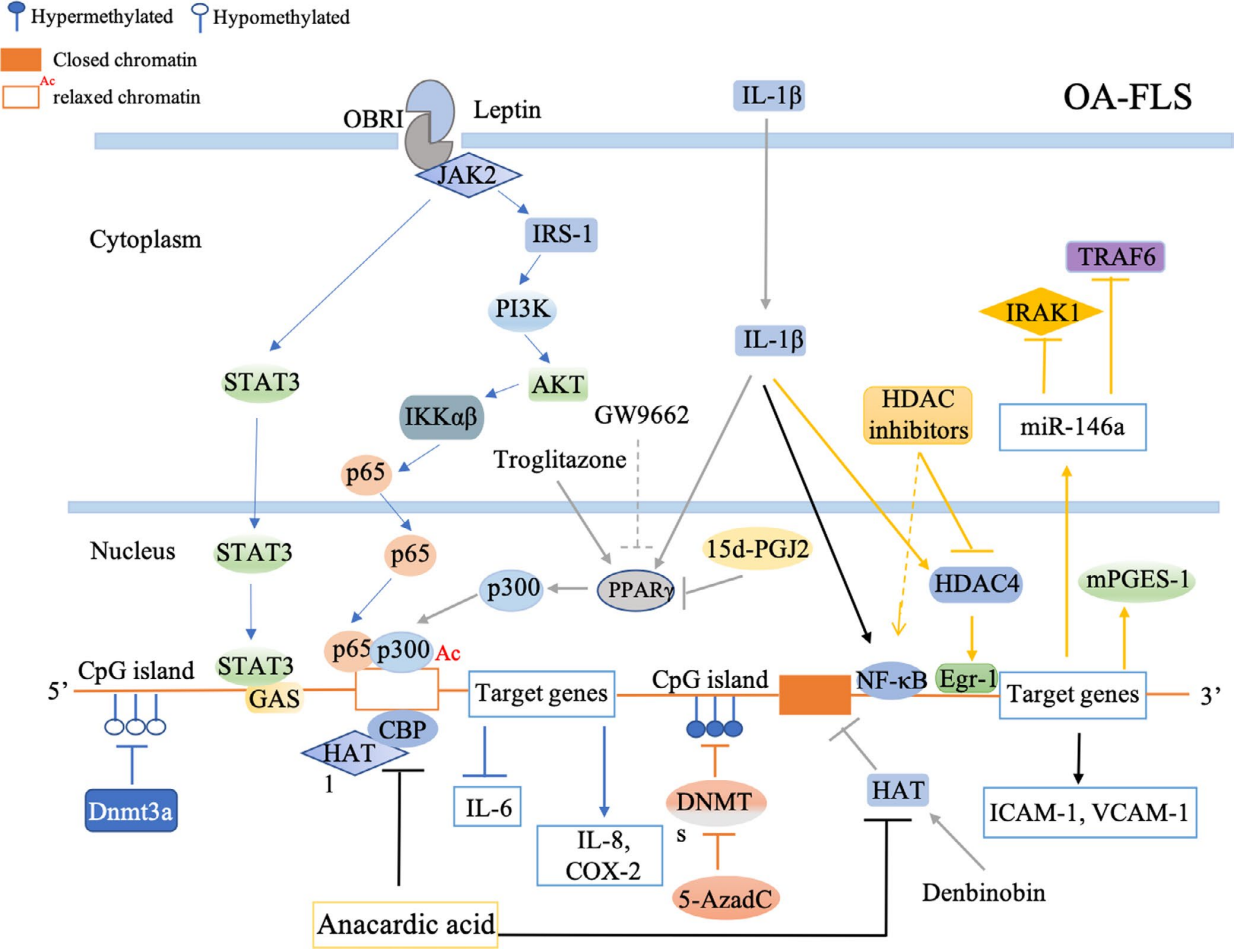
DNA methylation and histone acetylation involved in OA‐FLS inflammation. 5‐AzadC, a DNA methyltransferase inhibitor, up‐regulated miR‐146a by decreasing binding affinity between NF‐κB transcription factors and the hypermethylated miR‐146a regulatory regions. Dnmt3a, a DNA methyltransferase, increased the IL‐6 promoter methylation then suppressed IL‐6 production. Anacardic acid, a histone acetyltransferase inhibitor, decreased histone acetylation and binding of HAT1 and CBP on the IL‐6 promoter then suppressed IL‐6 expression. leptin promoted IL‐8 expression through the leptin receptor (OBRI), JAK2, STAT3 pathway as well as the initiation of IRSI, PI3K, Akt, NF‐κB‐dependent pathway and the following recruitment of p300. 15d‐PGJ2 inhibited IL‐1β‐induced COX‐2 production by suppressing H3 acetylation at the COX‐2 promoter through repressed HAT p300 recruitment. HDAC inhibitors (SAHA, LBH589) promoted the binding between the transcription factor NF‐κB and the miR‐146a promoter, subsequently increasing miR‐146a expression levels. Denbinobin increased miR‐146a expression, leading to a decreased monocyte adhesion by reducing the IL‐1β‐induced ICAM‐1, VCAM‐1 through regulated NF‐κB‐binding sites positioned within the miR‐146a promoter region. Anacardic acid attenuated denbinobin‐mediated increase in the HAT activity, histone H3 acetylation at both the NF‐κB‐binding sites and miR‐146a expression. HDAC4 promoted IL‐1‐induced mPGES‐1 production by up‐regulation of Egr‐1 transcriptional activity

Treatment of OA‐FLS with SAHA and LBH589 (two HDAC inhibitors) promoted the binding of the transcription factor NF‐κB to the miR‐146a promoter, subsequently increasing miR‐146a expression levels, and negatively regulating IRAK1/TRAF6 generation and IL‐6 production.^56^ Moreover, denbinobin (1,4‐phenanthrenequinone), a natural compound isolated from the stems of dendrobium moniliforme and ephemerantha lonchophylla, significantly increased miR‐146a expression, leading to decreased monocyte adhesion by reducing IL‐1β‐induced ICAM‐1 and VCAM‐1 through regulated NF‐κB‐binding sites positioned within the miR‐146a promoter region in OA‐FLS. Furthermore, anacardic acid treatment significantly attenuated the denbinobin‐mediated increase in the HAT activity, histone H3 acetylation at both the NF‐κB‐binding sites and miR‐146a expression, suggesting that denbinobin treatment up‐regulated miR‐146a expression.^10^ However, an increasing number of studies have reported that regulation of gene expression by DNA acetylation or deacetylation is dynamic and complex since HATs can serve as suppressers while HDACs can act as activators. For example, Nadir et al reported that HDAC inhibitors (trichostatin A, butyric acid and valproic acid) suppressed inflammatory cytokines induced microsomal prostaglandin E synthase‐1 (mPGES‐1) production at the transcriptional level in OA‐FLS. Moreover, overexpression of HDAC4, but not other HDACs, promoted mPGES‐1 promoter activation. Besides, HDAC4 was recruited to the mPGES‐1 promoter after stimulation with IL‐1 but without affected local deacetylation of histones H3 and H4. Furthermore, HDAC4 enhanced mPGES‐1 production by enhancing the activity of early growth response factor‐1 (Egr‐1), a main transcription factor in IL‐1‐induced mPGES‐1 production. These results suggest that HDAC4 up‐regulated IL‐1‐mediated mPGES‐1 production in OA‐FLS via Egr‐1 transcription activation^57^ (Figure 2).

Epigenetic modifications play a crucial role in the occurrence and development of OA. However, in recent years, the investigation of epigenetic modifications in OA development has been mainly focused on cartilage damage and chondrocytes,^58^, ^59^ and little attention has been paid to other cellular components in the synovium, especially OA‐FLS. Emerging evidence shows that synovitis is involved in the pathogenesis of OA. To our knowledge, DNA methylation and histone acetylation are two epigenetic modifications that have been studied in OA‐FLS. This neglected gap in OA‐FLS knowledge will be an important research field in the future and may provide new inspirations for the study of OA pathogenesis.

## 3 ACTIVATED SIGNALLING PATHWAYS IN OA‐FLS INFLAMMATION

### 3.1 NF‐κB pathway

Recently, a growing number of studies have revealed that abundant OA‐associated inflammatory factors play key roles in the inflammatory responses in OA‐FLS by activating the NF‐κB signalling pathway.

The connective tissue growth factor (CTGF), also referred to as CCN2, was found to be a pivotal inflammatory moderator.^60^, ^61^, ^62^, ^63^ Higher concentrations of CTGF in plasma and synovial fluid indicate a more severe disease status in patients with OA.^64^ Overexpression of CTGF in OA‐FLS has been reported to contribute to the chronic inflammatory environment by inducing the expression of IL‐6 through the α_ν_β_5_ integrin, the apoptosis signal‐regulating kinase 1 (ASK1), the p38/JNK and the activator protein 1 (AP‐1)/NF‐κB pathways.^60^ Moreover, in another study, CTGF and IL‐1β were found to synergistically augment the transcriptional activity of NF‐κB, accounting for the increased occurrence of IL‑1β‑mediated synovial inflammation in OA‐FLS.^61^ Berberine has been reported to reduce the production of CCN2‐induced IL‐1β in OA‐FLS and prevent cartilage damage in a collagenase‐induced OA (CIOA) through the α_v_β_3_/α_v_β_5_ integrins, reactive oxygen species (ROS), and the ASK1, p38/JNK, and NF‐κB signalling pathways.^63^ Furthermore, overexpressed CTGF could induce MCP‐1 production and recruit monocytes via the αvβ5 integrin, the focal adhesion kinase (FAK), methyl ethyl ketone (MEK), ERK and NF‐κB/AP‐1 signal transduction pathways in human OA‐FLS.^62^ The migrated and infiltrated monocytes in the inflamed areas have been proven to promote the generation of inflammation‐related factors and MMPs, which are the major aetiological factors in the development of OA.^65^

Follistatin‐like protein 1 (FSTL1) was originally acknowledged as a TGF‐β1‐inducible gene.^66^ The expression of FSTL1 mRNA and protein was found to be increased in the synovial tissue of OA patients. The serum and synovial fluid concentrations of FSTL1 were increased and served as a biomarker for the aggravation of the impaired joint in OA patients.^67^, ^68^ Although the function and the molecular mechanism of FSTL1 have not been fully elucidated, many investigations have demonstrated that FSTL1 is involved in FLS‐associated inflammation. For example, FSTL1 was found to act as a pro‐inflammatory protein by initiating the classical inflammation‐associated NF‐κB pathway and promoting FLS proliferation through the p53‐ and p21‐dependent pathways in human OA‐FLS.^69^ Moreover, p21 has been reported to block IL‐6 and MMP production in synovial FLS, which may further exacerbate the harmful effects of FLS in the pathogenesis of OA.^70^

**FIGURE 3:**
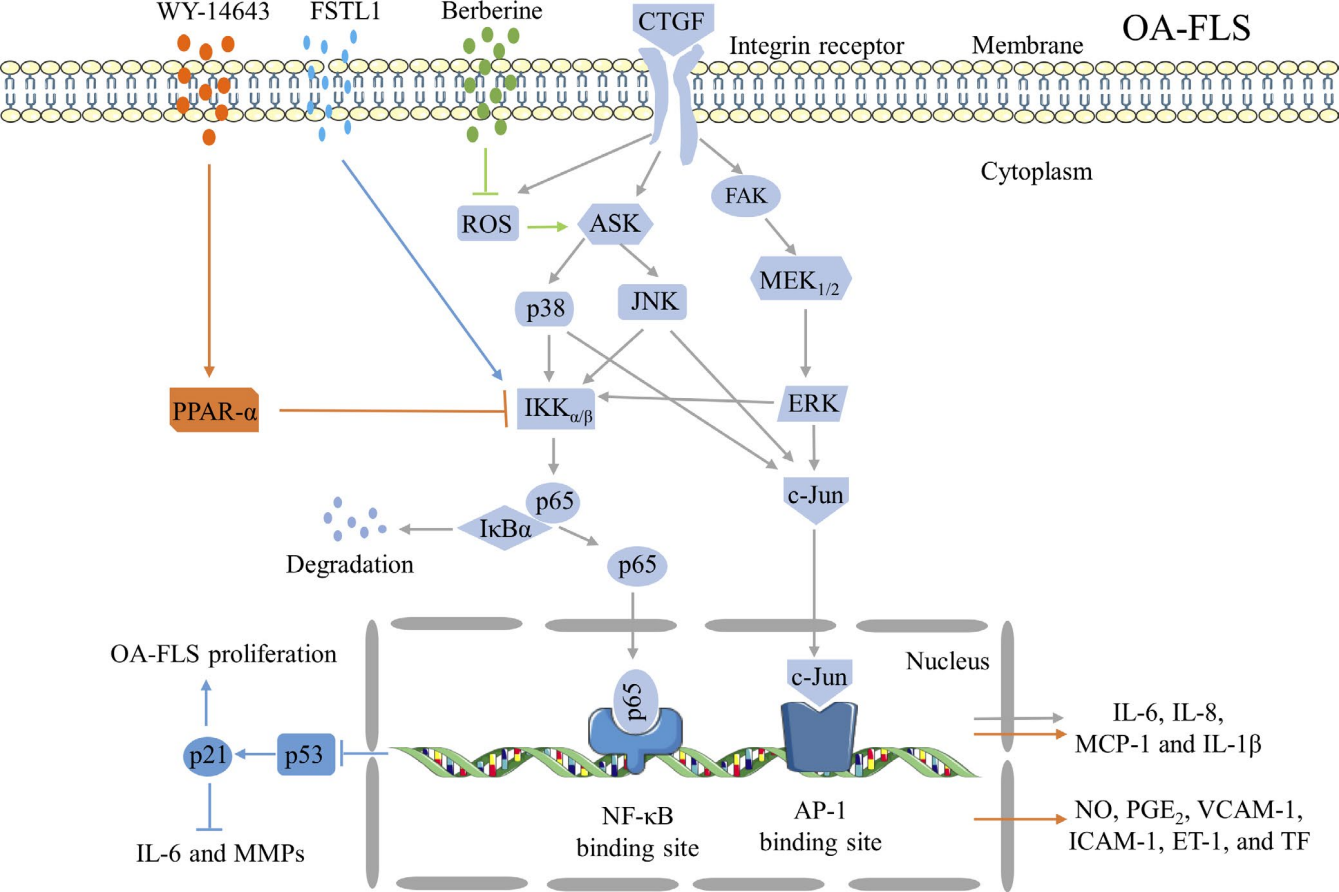
Activated NF‐κB signalling pathway in OA‐FLS inflammation. CTGF promoted the generation of IL‐6 in human OA‐FLS through the ανβ5 integrin, ASK1, p38/JNK and AP‐1/NF‐kB signal transduction pathways. CTGF‐induced IL‐β in human OA‐FLS through αvβ3/ανβ5 integrins, ROS, and ASK1, p38/JNK, and NF‐κB signal transduction pathways, which attenuated by berberine, an anti‐inflammatory isoquinoline alkaloid separated from the Chinese herb Rhizoma coptidis (Huang Lian). CTGF enhanced MCP‐1 production in human OA‐FLS through the ανβ5 integrin, FAK, MEK, ERK and NF‐κB/AP‐1 signalling pathway. FSLT1 elevated TNF‐α, IL‐1β and IL‐6 generation in human OA‐FLS via activated NF‐κB signalling pathway. FSLT1 increased OA‐FLS proliferation by down‐regulating p53 and p21. Moreover, p21 was reported to block the elevated IL‐6 and MMPs, participated in the development of OA. WY‐14643, an agonist of PPAR‐α, decreased LPS‐induced NO, PGE2, inflammatory mediators (VCAM‐1, ICAM‐1, ET‐1 and TF), and pro‐inflammatory cytokines (IL‐6, IL‐1β, TNF‐α and MCP‐1) production in human OA‐FLS by suppressing LPS‐mediated NF‐κB activation and IkB phosphorylation. p53, a tumour‐suppressor protein. p21, a cyclin‐dependent kinase inhibitor

The nuclear receptor peroxisome proliferator‐activated receptor alpha (PPAR‐α) has been described as an anti‐inflammatory regulator in both acute and chronic inflammation in some tissues, such as in the vascular wall, heart, nervous tissue, lungs, gut and liver.^71^ As a promising latent therapeutic target for inflammation‐associated disorders, the PPAR‐α agonist (WY‐14643) has been demonstrated to mitigate lipopolysaccharide (LPS)‐induced acute lung injury.^72^ In the pathogenesis of OA, the PPAR‐α agonist exerted a latent therapeutic effect, due to its potential anti‐inflammatory properties on chondrocytes,^73^ synovium^74^ and synovial FLS in OA.^75^ WY‐14643 decreased the generation of NO, PGE_2_, VCAM‐1, ICAM‐1, endothelin‐1 (ET‐1), tissue factor (TF), IL‐6, IL‐1β, TNF‐α and MCP‐1 in OA‐FLS, which were possibly highlighted because of their inhibitory effects on the nuclear translocation of NF‐κB.^75^, ^76^ Activation of PPAR‐α by WY‐14643 ameliorates the NF‐κB‐related inflammation, suggesting that this may be a promising therapeutic approach for treatment of the chronic inflammation in OA (Figure 3).

Other inflammation‐associated factors have also been reported to participate in the pathogenesis of OA through activated NF‐κB, such as the advanced glycation end products (AGEs), which decrease both the proliferation and the viability of OA‐FLS and up‐regulate the expression of pro‐inflammatory cytokines by activating the receptor for AGEs (RAGE) and the NF‐κB signalling pathway in human OA‐FLS.^77^ Overexpression of connexin43 (Cx43) has been detected in synovial biopsies of OA patients with an augmented amount of gap junction plaques.^78^ The elevated expression of the gap junction protein Cx43 was enough to increase the production of OA‐associated catabolic and inflammatory genes, such as IL‐1, IL‐6, MMP1, MMP13, a disintegrin and metalloproteinase with thrombospondin motifs 4 (ADAMTS4), and ADAMTS5. However, these pro‐catabolic and pro‐inflammatory effects could be reversed by a Cx43 knockout and NF‐κB inhibitors, suggesting that Cx43 participated in the pathogenesis of OA through activation of the NF‐κB signalling pathway.^79^ The gap junction inhibitors 18α‐glycyrrhetinic acid and octanol, significantly decreased the levels of MMPs in OA‐FLS and cultured FLS.^78^, ^79^

### 3.2 Wnt pathway

**FIGURE 4:**
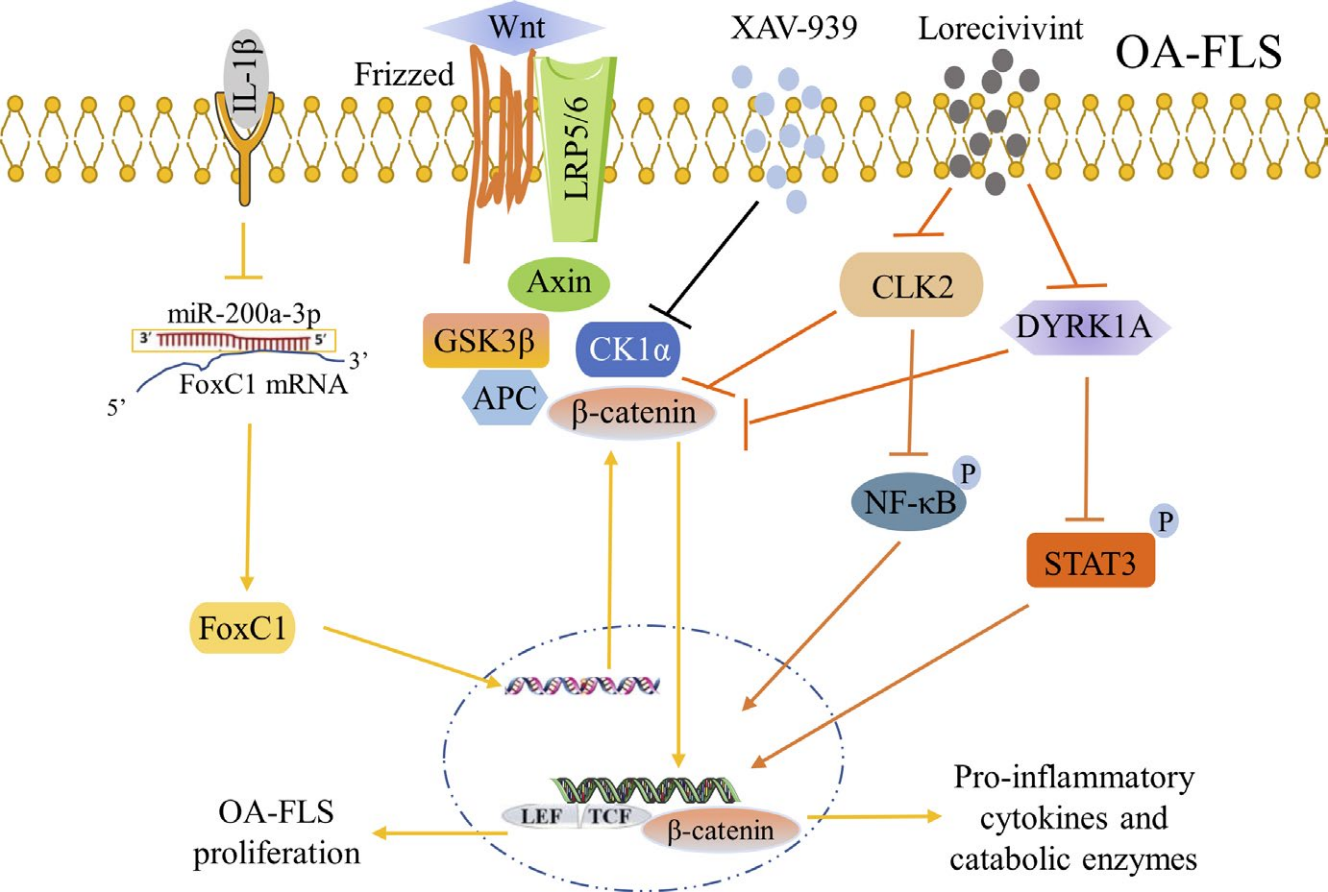
Wnt/β‐catenin signalling pathway in OA‐FLS inflammation. FoxC1, a target gene of miR‐200a‐3p, promoted human OA‐FLS proliferation, pro‐inflammatory cytokine and catabolic enzymes expression by elevated β‐catenin production. IL‐1β inhibited miR‐200a‐3p enhanced the effects of FoxC1 in human OA‐FLS. XAV‐939, a small‐molecule inhibitor of Wnt pathway, reduced proliferation and I collagen levels of OA‐FLS. Lorecivivint, a small‐molecule Wnt pathway inhibitor, declined pro‐inflammatory cytokines by inhibiting NF‐κB and STAT3 phosphorylation through reduced CLK2 and DYRK1A production in human OA‐FLS. LRP5/6: Lipoprotein receptor‐associated protein; GSK3β: Glycogen synthase kinase 3β; CK1: Casein kinase 1; APC: Adenomatous polyposis coli; LEF/TCF: Lymphoid enhancer factor/T‐cell; CLK2: CDC‐like kinase 2; DYRK1A: Dual‐specificity tyrosine phosphorylation‐regulated kinase 1A

Activation of the Wnt signalling pathway was not exclusive to chondrocytes but was also activated in articular FLS during the development of OA. Both β‐catenin and the forkhead box C1 (FoxC1) were elevated in OA synovial tissue and human OA‐FLS, and the highly activated β‐catenin was regulated by the overexpressed FoxC1. Moreover, FoxC1, the target gene of miR‐200a‐3p, which was negatively regulated by IL‐1β, increased the proliferation of OA‐FLS and the production of pro‐inflammatory cytokines through β‐catenin. FoxC1 and miR‐200a‐3p were involved in the IL‐1β‐induced inflammatory responses as IL‐1β‐induced expression of inflammatory cytokines could be reversed by FoxC1 knockout and miR‐200a‐3p overexpression in FLS. Furthermore, an intra‐articular injection of the FoxC1‐specific siRNA decreased the thickness of the synovium and the number of infiltrated immune cells, leading to an improvement of synovitis.^80^ Elevated canonical Wnt/β‐catenin signalling in the synovium was observed in destabilization of the medial meniscus (DMM) model of OA and human OA‐FLS. XAV‐939, a small‐molecule inhibitor of Wnt, mitigated OA severity through decreased synovitis and cartilage degeneration in vivo and significantly declined both the proliferation and concentrations of the type I collagen in OA‐FLS in vitro.^81^ Lorecivivint (SM04690), a first‐in‐class, reversible, ATP‐competitive, small‐molecule Wnt pathway inhibitor, inhibited NF‐κB and signal transducer and activator of transcription 3 (STAT3) production and abridged the IL‐6 and TNF‐α expression in a dose‐dependent manner, which was stimulated by IL‐1β and LPS in the synovial fibroblasts. Moreover, Lorecivivint inhibited a panel of inflammatory cytokines (IL‐1β, IL‐2, IL‐5, IL‐6, IL‐8, TNF‐α, interferon‐γ [IFNγ]) in the LPS‐stimulated synovial fibroblasts in vitro.^82^ These functions of Wnt contribute to the sophisticated inflammatory responses and proliferation of synovial fibroblasts in OA (Figure 4). At present, more attention needs to be focused on the treatment of bone erosion and cartilage destruction in the joints of OA patients. The Wnt pathway inhibitors XAV‐939 and lorecivivint have demonstrated comprehensive therapeutic effects in OA by promoting chondrogenesis, protecting cartilage and acting against inflammation. These comprehensive effects will potentially satisfy the urgent requirement for therapeutic strategies in OA rehabilitation. Therefore, the specific role of the Wnt pathway in OA and evaluation of therapeutic effects of Wnt pathway inhibitors on OA should be further evaluated.

The NF‐κB and Wnt pathways are the two most classical inflammation‐related signalling pathways, and they are the most studied signalling pathways in OA‐FLS and the pathogenesis of OA. Except NF‐κB and Wnt/β‐catenin signalling pathways, the mitogen‐activated protein kinase (MAPK) signalling pathway has also been relatively well studied in OA‐FLS as mentioned in different parts of this review. For example, TGF‐β1 increased anti‐inflammatory FOXO3 levels in OA‐FLS by suppressing miR‐92a via the AMPK and p38 pathways.^12^ Adipocytokines visfatin promoted IL‐6 production by activating the ERK/p38/JNK pathway.^33^ Moreover, there were some crosstalks between the MAPK and NF‐κB pathways in OA‐FLS‐mediated inflammation, such as CTGF inducing IL‐6 expression by the ASK1/p38/JNK/AP‐1/NF‐κB pathway^60^ and CTGF increasing MCP‐1 via the FAK/MEK/ERK, and NF‐κB/AP‐1 pathways in OA‐FLS.^62^ Besides, HMGB1 cooperated with IL‐1β increased ERK1/2 and p38 phosphorylation and a high dose of HMGB1 activated NF‐κB signalling.^83^ However, as we have mentioned before, the role of OA‐FLS in the development of OA has been neglected, and there are relatively few studies on other specific signalling pathways in OA‐FLS. For example, resistin increased MCP‐1 expression by activating the PI3K/AKT/mTOR pathway,^32^ but soya‐cerebroside reduced the IL‐1β‐induced MCP‐1 expression and monocyte migration by increasing the miR‐432 generation through the AMPK and AKT pathways.^35^ Nowadays, emerging evidence has provided insights into the pathogenic mechanisms underlying the development of synovial inflammation in OA. It is hoped that more studies will focus on the role of inflammatory associated signalling pathways in OA‐FLS in the future.

## 4 OTHER IMPORTANT ASSOCIATED FACTORS IN OA‐FLS INFLAMMATION

### 4.1 High mobility group box 1 (HMGB1)

The HMGB1, a nuclear DNA‐binding protein, can be negatively released by necrotic cells or secreted by myeloid cells in response to LPS or pro‐inflammatory cytokine stimulation.^84^ Several important receptors of HMGB1 have been identified, including the receptor for advanced glycated end products (RAGE) and Toll‐like receptor (TLR) 2/4 which are inflammatory associated receptors.^85^

The expression HMGB1 and its receptors (RAGE, TLR2, TLR4) were up‐regulated in OA.^83^, ^85^, ^86^ Overexpressed HMGB1 participated in OA development by interacting with its receptors, resulting in the production of MMPs and pro‐inflammatory cytokines. HMGB1 promoted both the mRNA and protein expression of RAGE and enhanced its cell surface expression in OA‐FLS. Moreover, HMGB1 interacted with RAGE and activated c‐Src and AKT, accounting for the transactivation of IL‐6 expression in OA‐FLS.^1^ Besides, activated RAGE in OA‐FLS facilitated MMP and type II collagen production.^87^ These results indicate that the HMGB1‐RAGE complex is involved in OA development and the up‐regulated AGEs partially explain how elderly populations are more sensitive towards OA. In addition to RAGE, HMGB1 have been reported to bind to TLRs. HMGB1 interacts with TLR2 and TLR4, leading to up‐regulate NF‐κB and initiation of the synthesis and release of pro‐inflammatory cytokines.^88^ However, Heidi et al reported that HMGB1 in complex with LPS or IL‐1 promoted the production of TNF, IL‐6 and IL‐8 and HMGB1 in complex with IL‐1β elevated MMP expression in OA‐FLS via IL‐1RI but not TLR4.^89^ Nowadays, almost all studies of the interaction between HMGB1 and TLR are performed in OA chondrocytes. In the future, the role of the HMGB1‐TLR complex should also be investigated in OA‐FLS.

In addition to receptor binding, HMGB1 also exhibits a pro‐inflammatory effect by potentiating the pro‐inflammatory effects of cytokines or forming complexes with cytokines in the development of OA. The increase in the levels of both HMGBI and RAGE is closely related to the progression of OA. Overexpressed HMGB1 strengthened the pro‐inflammatory effects of IL‐1β in OA‐FLS.^90^ Isabel et al reported HMGB1 accompanied by IL‐1β significantly increased both the mRNA and protein expression of IL‐6, IL‐8, CCL2, CCL20, MMP‐1 and MMP‐3 but no affect was obtained without IL‐1β in OA‐FLS.^83^ Moreover, the cooperation of HMGB1 and IL‐1β was followed by increased ERK1/2 and p38 phosphorylation and a high dose of HMGB1 activated NF‐κB signalling.^83^ It has also been reported that an intra‐articular expression of HMGB1 exacerbated synovitis in mice, while neutralized HMGB1 inhibited the progression of experimental arthritis.^91^ In addition, HMGB1 in a complex with LPS or IL‐1 promoted an enhanced inflammatory phenotype in OA‐FLS.^89^, ^92^ Noteworthy, haem oxygenase‐1 (HO‐1) was found to decrease the production of HMGB1 and MMPs in OA‐FLS, suggesting that HO‐1 may be a promising therapeutic strategy to reduce inflammation‐associated factors and degradation‐related MMPs in the development of OA.^93^

### 4.2 Nerve growth factor (NGF)

The NGF, a member of the neurotrophin family, has many biological functions related to inflammation and immune responses. Its expression levels were found to be increased after stimulation with IL‐1β and TNF‐α.^94^, ^95^ It has been confirmed that NGF exerts an anti‐inflammatory role in numerous inflammatory disorders and animal models.^96^, ^97^ Moreover, NGF was found to be overexpressed in human OA‐FLS both under normal conditions and after stimulation with pro‐inflammatory cytokines. However, elevated NGF levels reduced the IL‐1β‐induced TNF‐α and inducible nitric oxide synthase (iNOS) generation and played a protective role rather than an pro‐inflammatory role in OA‐FLS.^13^ Moreover, NGF and its receptors, NGF‐R, a TrkA (tyrosine kinase) receptor with a high affinity and the p75 neurotrophin receptor, with a low‐affinity for NGF, were up‐regulated by pro‐inflammatory cytokines in OA‐FLS. Overexpression of NGF was found to stimulate the proliferation of OA‐FLS in a dose‐dependent manner, which may aggravate the musculoskeletal pain, disability and hyperplastic synovium in OA.^98^ Pain is the most noticeable clinical symptom in OA patients; therefore, extenuating pain by targeting synovial levels of the axonal product‐promoting factors, particularly NGF and TrkA, should be a priority of OA therapy as this may considerably reduce disability and improve the quality of life of OA patients.

### 4.3 Bradykinin (BK)

BK is a pro‐inflammatory nonapeptide vasodilator (H‐Arg‐Pro‐Pro‐Gly‐Phe‐Ser‐Pro‐Phe‐Arg‐OH) which is produced by kininogen precursors in the plasma and interstitial fluids. It has been reported that BK is produced in the synovial fluid of OA patients, and its level in synovial fluid correlates with biochemical markers of cartilage erosion and inflammation in the OA knee.^99^ Recently, there has been increasing focus on the important role of BK in the pathophysiology of OA. Bradykinin in human FLS has been reported to induce the production of inflammatory‐related factors, for example, prostaglandins, cytokines and chemokines, which participate in synovial inflammation.^11^ Moreover, BK elevated the expression of PGE2 and cyclooxygenase (COX)‐2 and these effects could be enhanced by IL‐1β in FLS. It has also been reported that pre‐treatment with fasitibant (MEN16132), a high‐affinity and selective BK receptor antagonist, could completely inhibit the synergistic effect of BK and IL‐1β in FLS.^100^

### 4.4 Transient receptor potential ankyrin 1 (TRPA1)

The TRPA1, a calcium‐permeable ion channel, was found to be highly expressed in neuronal cells, where it regulated pain and inflammation.^101^ Besides acting as a nociceptor, TRPA1 expression has also been observed in non‐neuronal cells, such as synoviocytes^102^ and lymphocytes,^103^ indicating its significant role in inflammation. Moreover, TRPA1 was elevated in the synovial tissue of OA rats,^104^ while TRPA1 deficiency protected mice from inflammation, cartilage deterioration, swelling joints and pain, in experimental animal models of OA. It has also been reported that a TRAP1 knockout, or pharmacological inhibition with TCS5861528, decreased the IL‐1‐induced cyclooxygenase‐2 (COX‐2) production and reduced the IL‐1β‐induced apoptosis in chondrocytes of OA.^105^ In addition, TRAP1 was expressed in human OA‐FLS and its expression could be increased by LPS in a time‐ and dose‐dependent manner, which subsequently intensified the Ca^2+^ influx in OA‐FLS. Pharmacological suppression and genetic silencing of TRPA1 reduced IL‐1β, TNF‐α, IL‐6 and MMP production in the LPS‐treated human OA‐FLS.^106^ All the above‐mentioned evidence suggests that neutralizing TRAP1 may lead to anti‐inflammatory and cartilage protective effects in OA, providing a novel and comprehensive treatment target for OA.

### 4.5 Chitosan oligosaccharide (COS)

COS, derived from chitosan by enzymatic hydrolysis, is composed of β‐1‐4‐linked D‐glucosamine.^107^ Owing to its anti‐apoptotic and chondro‐protective characteristics, achieved via regulating the p38 MAPK signalling pathway, COS has been demonstrated to have potential as a OA treatment.^108^ Moreover, COS increased the expression of osteoprotegerin (OPG) and decreased the expression of receptor activator of the NF‐κB ligand (RANKL).^109^ In addition to its protective effect on cartilage, COS was also shown to have powerful anti‐inflammatory effects by activating AMPK and extenuating inflammatory responses in OA‐FLS. COS inhibited TNF‐α‐regulated iNOS and COX‐2 generation by activating AMPK in both human and rabbit OA‐FLS^110^ (Table 1).

**TABLE 1:** Inflammation‐associated factors in OA‐FLS inflammation

Mediators	Functions	Description of evidences	References
HMGB1	Pro‐inflammation	Transactivated of IL‐6 expression in OA‐FLS	[1]
		Enhanced the pro‐inflammatory effects of IL‐1β and LPS in OA‐FLS	[89, 90, 92]
NGF	Anti‐inflammation and algogenic	Reduced IL‐1β‐induced TNF‐α and iNOS generation in OA‐FLS	[13]
BK	Pro‐inflammation and algogenic	Induced prostaglandins, cytokines, and chemokines production in human FLS	[11]
		Elevated the expression of PGE2, COX‐2 inhuman FLS	[100]
TRPA1	Pro‐inflammation	Promoted IL‐1β, TNF‐α, IL‐6 and MMPs production in LPS‐treated OA‐FLS	[106]
COS	Anti‐inflammation	Inhibited TNF‐α‐regulated iNOS and COX‐2 generation by activating the AMPK in both human and rabbit OA‐FLS	[110]

## 5 CLINICAL TRIALS

Owing to the absence of structurally‐improved drugs, the traditional treatment of OA is limited to the relief of pain and symptoms. The eventual joint replacement for patients with severe conditions and those who are insensitive to traditional medicines is inevitable. However, there is a huge treatment gap between pain relief and surgery. Therefore, there are two approaches in the development of OA medications before surgery. One approach focuses on relieving pain in patients with OA, while the other aims to develop structurally improved drugs that directly address the structural improvement needs of OA drugs.

Clinical trials evaluating pain relief treatment interventions are based on the above‐mentioned factors. Abundant clinical trials have evaluated anti‐NGF agents in different phases, including tanezumab (phase Ⅲ), fasinumab (phase Ⅲ), fulranumab (abandoned in phase Ⅲ not due to safety issues but due to phase Ⅲ clinical trial investment/funding issues), ABT‐110 (abandoned in phase Ⅰ) and MEDI‐578 (abandoned in phase Ⅰ). Tanezumab was the first agent to enter clinical trials and now remains a pacemaker in the development of anti‐NGF antibodies, although it was suspended by the United States Food and Drug Administration (USFDA) twice due to associations with a rapidly progressive OA and side effects such as a sympathetic neuron damage. Recently, phase Ⅲ clinical trials of tanezumab have demonstrated its safety and effectiveness (NCT02709486, NCT02697773, NCT02528188). As recently announced by Pfizer and Lilly, the USFDA has accepted the biologics licence application (BLA) of monoclonal antibody tanezumab (2.5 mg subcutaneously [SC]). The agent is currently being evaluated for patients with chronic pain caused by moderate‐to‐severe OA whose pain cannot be adequately relieved by other analgesics. Tanezumab is the first fast‐track NGF inhibitor, and if approved, it will be a first‐in‐class treatment for OA pain. Its only competitor, fasinumab (NCT03285646), has been demonstrated to significantly reduce the painful symptoms. However, the development of fasinumab has been halted by the FDA following an adverse event in which high doses of fasinumab were found to aggravate OA. While the efficacy of various fasinumab regimens will be tested in the future, it is now being developed at a lower dose.

Clinical trials evaluating structurally improved drugs are based on the previously mentioned mediators. Activation of the Wnt signalling pathway has been reported in the development of OA; therefore, inhibiting the activated Wnt pathway may be an effective way for a symptomatic OA treatment. Preclinical studies suggested that SM04690 promoted the production of new articular cartilage, reduced cartilage degeneration and joint inflammation by inhibiting the cdc‐like kinase 2 (CLK2) and the dual‐specificity tyrosine kinase (DYRK1A).^82^ Clinical trials (NCT02095548, NCT02536833, NCT03122860) reported that SM04690 was safe, well tolerated, relieved the pain, increased the medial joint space width (mJSW) and improved the symptoms in the treatment of OA. Now the phase Ⅱ (NCT03706521) and phase Ⅲ (NCT03928184) clinical trials of lorecivivint are recruiting volunteers.

In the clinical trial NCT02964624, which is now recruiting, the levels of HMGB1 in serum will be evaluated as a biomarker of OA and is related to the functional capacity of knee joints in patients who suffer from OA after rehabilitation protocol. HMGB1 was reported to be significantly elevated in OA and to participate in the development of OA by releasing abundant pro‐inflammatory cytokines. The phase Ⅱ clinical trials (NCT01091116) of the bradykinin receptor antagonists MEN16132 in the treatment of OA were completed in 2013. The phase II clinical trials (NCT02205814) of fasitibant, another name for MEN16132, were completed in 2015 (Table 2).

**TABLE 2:** Clinical trials based on inflammation‐related‐factors in OA‐FLS inflammation

Mediators	Names and mechanisms	NCT numbers	Study titles	Phases	States
Wnt		NCT03727022	A study evaluating the safety, tolerability and efficacy of two injections of SM04690 for the treatment of moderately to severely symptomatic knee osteoarthritis.	Ⅱ	Active, not recruiting
	Lorecivivint (Wnt pathway inhibitor)	NCT03706521	A study utilizing imaging techniques and Evaluating the safety and efficacy of SM04690 for the treatment of moderately.	Ⅱ	Recruiting
		NCT03928184	A study utilizing patient‐reported and radiographic outcomes and evaluating the safety and efficacy of lorecivivint (SM04690) for the treatment of moderately to severely symptomatic knee osteoarthritis (STRIDES‐X‐ray).	Ⅲ	Recruiting
HMGB1	None	NCT02964624	Osteoarthritis biomarkers and rehabilitation (Knee).	Not Applicable	Recruiting
NGF	Tanezumab (Anti‐NGF)	NCT02697773	Efficacy and safety of a subcutaneous tanezumab titration dosing regimen in subjects with moderate‐to‐severe osteoarthritis of the hip or knee.	Ⅲ	Completed Has results
	Fasinumab (Anti‐NGF)	NCT03285646	Evaluate the efficacy and safety of fasinumab in patients with moderate‐to‐severe chronic low back pain and osteoarthritis of the hip or knee.	Ⅲ	Completed
	LEVI‐04 (Anti‐NGF)	NCT03227796	Safety, tolerability & pharmacokinetics of LEVI‐04 in healthy volunteers and patients with osteoarthritis knee pain.	Ⅰ	Recruiting
BK	Fasitibant (B2‐receptor antagonist)	NCT01091116	A locally injected bradykinin antagonist for treatment of osteoarthritis (ALBATROSS).	Ⅱ	Completed Has results
		NCT02205814	Fasitibant intra‐articular injection in patients with symptomatic osteoarthritis of the knee (ALBATROSS‐3).	Ⅱ	Completed Has results
	Icatibant (B2‐receptor antagonist)	NCT00303056	Efficacy and safety study of intra‐articular multiple doses of icatibant in patients with painful knee osteoarthritis.	Ⅱ	Completed

## 6 DISCUSSION AND FUTURE PERSPECTIVE

Although it has been recognized that the primary symptom of OA is caused by the barely functional chondrocytes, recent investigations into the pathogenesis of OA has indicated that the inflammation in the synovial tissue plays an important role in the patho‐mechanism of OA. The synovium released lubricin and hyaluronic acid are very important in decreasing articular surface friction, protecting cartilage surface and maintaining the homeostasis of cartilage.^111^ However, in the development of OA, inflammation is another key feature of OA synovium. Specifically, they include a range of abnormalities, such as synovial hyperplasia, macrophage and lymphocyte infiltration, neovascularization and fibrosis. The changed synovium produces less lubricin and hyaluronic acid as well as loses the ability of retention of lubricin and hyaluronic acid and the ability of preventing plasma from entering and depositing on the articular surface, leading to articular wear and ultimately cartilage degradation. Targeting synovial inflammation might slow down the pathological change of synovium and protect cartilage from degradation. Besides, there is no intrinsic vascular or lymphatic supply in articular cartilage, and therefore, its nutrients are derived from synovial fluid secreted by synovium and other adjacent tissue.^112^ During inflammation, synovium was induced to secrete numerous soluble catabolic and pro‐inflammatory regulators, such as cytokines, MMPs, NO, PGE2 and neuropeptides. These regulators aggravate the inflammatory response in the synovium and diffuse through the synovium into the synovial fluid promoting the degradation of cartilage. Targeting synovial inflammation might suppress catabolic and pro‐inflammatory mediators secreted by the synovial membrane in synovial fluid. Cartilage damage, in turn, exacerbates inflammation in the synovium, resulting in a vicious cycle.^113^ Moreover, previous research has described that the synovial inflammation in OA was recognized to be a result of underlying articular breakdown, with the synovial inflammation derived from macrophage phagocytosis of joint cartilage or bone particles, calcium pyrophosphate dihydrate or calcium hydroxyapatite crystals with increased catabolic mediators.^114^, ^115^ However, recent articles reported that synovial inflammation plays a role in the early disease and might be a precursor of OA, rather than cartilage damage,^15^ since synovial inflammation was present in all states of OA,^116^ even severe in early OA.^6^ Furthermore, Atukorala et al reported that synovial inflammation correlated with the probability of emerging incident radiographic knee osteoarthritis (ROA) and was more obvious in the year before the development of ROA.^15^ Recently studies about synovial inflammation and the pathogenesis of OA indicated that synovial inflammation is no longer recognized as a bystander and might precede cartilage damage as a pivotal focus. Therefore, targeting synovial inflammation in early OA might be a promising approach to slowing down the progression of OA and perhaps even prevent articular cartilage destruction.

Although OA is one of the most common joint diseases in ageing people, no drug has been approved for the curative treatment of OA as cartilage has a limited self‐repair ability. Analgesics and anti‐inflammatory medicines are the prevailing OA treatments for either pain relief or symptom reduction, but they do not address the cause of the disease and are sometimes accompanied by adverse effects. Targeting synovial inflammation is a potential target for future development of disease‐modifying OA drugs (DMOADs).^117^ Nowadays, the ‘magic bullet’ in the treatment of OA should not only mitigate pain and alleviate symptoms but also improve the condition of the destroyed cartilage. This should be the main trends of OA drug research and development in the future. However, it is difficult for a single agent to relieve pain, alleviate symptoms and reverse the damage to cartilage. Thus, a combination of pain relief and DMOADs will be a promising strategy for OA treatment.

OA occurs in weight‐bearing joints and with a lack of early diagnostic indicators, and patients with early OA usually consume some painkillers and/or do not even realize they have OA, thus missing the best treatment opportunity at this early stage. Moreover, synovial inflammation predates cartilage damage and coexists throughout the course of OA. An early diagnosis and targeting early synovial inflammation may be a promising therapeutic strategy for alleviating the symptoms and reducing progression of OA, perhaps even preventing its occurrence. In the future, more investigations should illustrate the precise role of synovial inflammation in the pathogenesis of OA and the relationship between synovial inflammation and cartilage damage.

## CONFLICT OF INTEREST

The authors have declared no conflicts of interest.

DATA AVAILABILITY STATEMENT

No new data generated.

## AUTHOR CONTRIBUTION

**Dafei Han:** Data curation (equal); Writing‐original draft (equal). **Yilong Fang:** Data curation (equal); Writing‐original draft (equal). **Xuewen Tan:** Writing‐review & editing (equal). **Haifeng Jiang:** Writing‐review & editing (equal). **Xun Gong:** Writing‐review & editing (equal). **Xinming Wang:** Writing‐review & editing (equal). **Wenming Hong:** Writing‐review & editing (equal). **Jiajie Tu:** Conceptualization (equal); Writing‐review & editing (equal). **wei wei:** Writing‐review & editing (equal).
